# D2 Partial Agonists vs Full Antagonists in Young Adults with Early SMI and Co-Occurring Substance Use

**DOI:** 10.1192/j.eurpsy.2026.10486

**Published:** 2026-07-31

**Authors:** C. Noureddine, C. Wang, N. Burlant, N. Ahuja, S. Lynch, A. Sterchele

**Affiliations:** 1 Psychiatry, Icahn School of Medicine at Mount Sinai; 2 Addiction Psychiatry, Columbia University, New York, United States

## Abstract

**Introduction:**

Roughly one in four to one in two individuals with serious mental illness (SMI) also have a co-occurent substance use disorder (SUD). Often times, during inpatient hospitalization, the treatment of the underlying psychiatric disorder such as schizophrenia or bipolar disorder takes priority over treating the SUD. In this study, we wanted to assess if among patients with SMI who are early in their illness and have co-occurring SUD, there are any differences between those who receive partial agonist vs full D2-antagonist long-acting injectable antipsychotics (LAIs).

**Objectives:**

Elucidate the sociodemographic and clinical characteristics that contribute to choosing a D2 partial agonist vs a full antagonist LAI

Identify the impact of a D2 partial agonist vs a full antagonist LAI on hospital readmission on SMI patients with SUD

**Methods:**

This IRB-approved retrospective chart review examined 106 psychiatric inpatients across three different New York City-based inpatient units for patients ≤25 years old with ≤2 hospitalizations and co-morbid substance use who received either a partial agonist (aripiprazole) (N=31) or a full antagonist (risperidone, paliperidone, haloperidol, fluphenazine) (N=75) LAI. T-tests and chi-squared tests were calculated to compare sociodemographic and clinical characteristics, and linear regression was used to identify independent predictors of time to readmission. Effect sizes were calculated for statistically significant results.

**Results:**

Patients who received a partial agonist LAI were less likely to have public insurance (χ2 = 7.06, df = 1, p < 0.01, V = 0.26), less likely to be Black and more likely to be White or Asian (χ2 = 10.65, df = 4, p = 0.03, V = 0.32), and had on average fewer prior psychiatric emergency room visits (t = 2.02, df = 104, p = 0.05, d = 0.35). They were more likely to present for suicidality and less likely to present for psychosis (χ2 = 12.33, df = 2, p < 0.01, V = 0.34). Partial agonists had a lower rate of adverse reactions (χ2 = 5.34, df = 1, p = 0.02, V = 0.22). A linear regression model was developed using significant results from above and found that the only independent predictor of time to readmission was receiving a partial agonist vs full antagonist LAI, which predicted a delay in time to readmission of 135 days (95% CI 35.36-234.16, p = 0.01).

**Conclusions:**

In patients with early SMI and substance use, D2 partial agonists may be more frequently used for primary mood disturbance or suicidal behavior rather than psychosis, and full D2 antagonists may be more often prescribed to those with greater history of contact with psychiatric emergency services. Further, D2 partial agonists may be better tolerated in this population compared to full antagonists. Most importantly, receiving a partial agonist LAI may increase time to readmission.

**Disclosure of Interest:**

None Declared

